# Comparative costs and activity from a sample of UK clinical trials units

**DOI:** 10.1186/s13063-017-1934-3

**Published:** 2017-05-02

**Authors:** Daniel Hind, Barnaby C. Reeves, Sarah Bathers, Christopher Bray, Andrea Corkhill, Christopher Hayward, Lynda Harper, Vicky Napp, John Norrie, Chris Speed, Liz Tremain, Nicola Keat, Mike Bradburn

**Affiliations:** 1 CTRU, Regent Court, 30 Regent Street, Sheffield, S1 4DA UK; 2 Clinical Trials and Evaluation Unit, School of Clinical Sciences, University of Bristol, Queens Building, Level 7, Bristol Royal Infirmary, Bristol, BS2 8HW UK; 3 Caudwell Children, Minton Hollins, Shelton Old Road, Stoke on Trent, Staffordshire, ST4 7RY UK; 4 Diabetes Trials Unit, OCDEM, Churchill Hospital, Old Road, Oxford, OX3 7LJ UK; 5 University of Southampton, Clinical Trials Unit, MP131, Southampton General Hospital, Tremona Road, Southampton, Hants SO16 6YD UK; 6 Peninsula Clinical Trials Unit, Peninsula College of Medicine & Dentistry, Room N14, ITTC Building 1, Tamar Science Park, Plymouth, Devon PL6 8BX UK; 7 MRC Clinical Trials Unit at UCL, Aviation House, 125 Kingsway, London, WC2B 6NH UK; 8 Clinical Trials Research Unit, University of Leeds, Leeds, LS2 9JT UK; 9 Centre for Healthcare Randomised Trials (CHaRT) Health Services Research Unit, University of Aberdeen, 3rd Floor, Health Sciences Building Foresterhill, Aberdeen, AB25 2ZD UK; 10 Newcastle Clinical Trials Unit, Newcastle University, 1-4 Claremont Terrace, Newcastle upon Tyne, NE2 4AE UK; 11 National Institute for Health Research, Evaluation, Trials and Studies Coordinating Centre, University of Southampton, Alpha House, Enterprise Road, Southampton, SO16 7NS UK; 12 Cancer Research UK, Angel Building, 407 St. John Street, London, EC1V 4AD UK

## Abstract

**Background:**

The costs of medical research are a concern. Clinical Trials Units (CTUs) need to better understand variations in the costs of their activities.

**Methods:**

Representatives of ten CTUs and two grant-awarding bodies pooled their experiences in discussions over 1.5 years. Five of the CTUs provided estimates of, and written justification for, costs associated with CTU activities required to implement an identical protocol. The protocol described a 5.5-year, nonpharmacological randomized controlled trial (RCT) conducted at 20 centres. Direct and indirect costs, the number of full time equivalents (FTEs) and the FTEs attracting overheads were compared and qualitative methods (unstructured interviews and thematic analysis) were used to interpret the results. Four members of the group (funding-body representatives or award panel members) reviewed the justification statements for transparency and information content. Separately, 163 activities common to trials were assigned to roles used by nine CTUs; the consistency of role delineation was assessed by Cohen’s *κ*.

**Results:**

Median full economic cost of CTU activities was £769,637 (range: £661,112 to £1,383,323). Indirect costs varied considerably, accounting for between 15% and 59% (median 35%) of the full economic cost of the grant. Excluding one CTU, which used external statisticians, the total number of FTEs ranged from 2.0 to 3.0; total FTEs attracting overheads ranged from 0.3 to 2.0. Variation in directly incurred staff costs depended on whether CTUs: supported particular roles from core funding rather than grants; opted not to cost certain activities into the grant; assigned clerical or data management tasks to research or administrative staff; employed extensive on-site monitoring strategies (also the main source of variation in non-staff costs). Funders preferred written justifications of costs that described both FTEs and indicative tasks for funded roles, with itemised non-staff costs. Consistency in role delineation was fair (*κ* = 0.21–0.40) for statisticians/data managers and poor for other roles (*κ* < 0.20).

**Conclusions:**

Some variation in costs is due to factors outside the control of CTUs such as access to core funding and levels of indirect costs levied by host institutions. Research is needed on strategies to control costs appropriately, especially the implementation of risk-based monitoring strategies.

**Electronic supplementary material:**

The online version of this article (doi:10.1186/s13063-017-1934-3) contains supplementary material, which is available to authorized users.

## Background

The increasing costs of clinical trials, of great concern a decade ago [1, 2], continue to be the subject of comment and debate worldwide [3–11] despite some indications of freezes or cuts in funding since the onset of the global recession in 2008 [12, 13]. The costs and delays associated with a steady increase in bureaucracy in Europe and America are well-attested [14–30]. The extent to which increasing trial complexity has been responsible for driving up costs has also been the subject of scrutiny, with documented increases in the numbers of trial processes and eligibility criteria [31–33]. Some claim that incremental increases in complexity are not always matched by a corresponding increase in staffing [33–35], and that this can lead to staff burnout and attrition as well as challenges associated with crisis management [34, 36].

Clinical trials units (CTUs) are specialised research entities that may assist with the design and central coordination of trials [37]. The case has been made for CTUs being central to the maintenance of quality, credibility and impact of clinical trials [38, 39], and their costs are already the subject of research publications [37, 39]. Previous work has identified research activities associated with clinical trials and the key determinants of their cost [37]. The purpose of the present work is to provide evidence of, and rationales for, variations in the cost of those activities between units based on the experiences of ten UK CTUs.

## Methods

Between March 2013 and June 2014 the authors pooled their experiences at four 2-h teleconferences, two full-day workshops and intermittent e-mail contact. Three more formal pieces of research were also designed, to help the group to pool their experiences and expertise. One investigated variation in costs of CTU-based activities, a second involved written justifications of the costs, and a third considered differences in role delineation across CTUs.

### Variation in costs

CTUs based in higher education institutions (HEIs) volunteered to cost CTU activities for the same protocol given in vignette form (Additional file 1). CTUs were asked to budget only for their own research costs, that is, including monitoring carried out by the CTU, but excluding research activities carried out by centres such as the collection and entry of data by research nurses. The protocol specified a 66-month surgical trial, timetabled to run between 1 January 2015 and 30 June 2020 with 12 months’ set-up, 24 months’ recruitment, 24 months’ follow-up and 6 months’ close-out. It assumed that four centres would open each month between months 8 and 12 and the first operations would be carried out on randomised participants between months 13 and 17. Centres were projected to identify on average 1.3 eligible participants per centre, per month, of whom 45% would consent.

The direct costs of implementing a trial by a CTU are conventionally divided into staff and non-staff costs. Salary details reported by CTUs were institution-specific for the financial year 2013–2014; profiles of staff grades and spine points were chosen by the CTUs undertaking the exercise; professorial salaries can vary substantially (£60,000 to £110,000). Summary costs were tabulated using the Transparent Approach to Costing (TRAC) categories [40]. In the UK, staff costs are currently classified as directly allocated or directly incurred (representing services incurred or purchased specifically for a project). Increasingly, CTUs are hosted by HEIs and directly allocated costs will typically cover Higher Education Funding Council for England (HEFCE-)funded university staff who would otherwise be deployed on legitimate HEI activities such as teaching. Indirect and estate costs (collectively referred to as indirect below) are defined as those ‘not directly related to any one project or activity, but [which] are a necessary part of the costs of undertaking an activity’ [40]. For a UK CTU hosted by an HEI, the indirect costs associated with a research project are related to their HEI’s infrastructure; they are typically calculated in relation to the number of researcher full time equivalents (FTEs) (but not technical or administrative staff) funded by the project. (NHS institutions cannot charge indirect costs, only 100% of directly incurred costs; none of the CTUs taking part costed for NHS staff).

Telephone interviews with CTU representatives who performed the costings were conducted by the lead author to elicit data on local practices and reasons why they might produce costs that differed from those of other CTUs. We had few preconceived opinions about reasons for varying costs and we used unstructured interviews to collect data, with no theoretical framework for its analysis [41, 42]. There were no topic guides, with the cost-specifications themselves forming the basis for questions and discussion. Interviews, which lasted between 30 and 60 min, were digitally recorded and transcribed verbatim, with themes derived inductively by the interviewer. Key themes which arose during minuted meetings held between January 2013 and June 2014 were also incorporated into the results and discussion. Because all interviewees were committee members and coauthors, we considered the work to be exempt from ethical approval and consent to be implied.

### Justification of costs

CTUs that submitted costs were also asked to submit justification of their costs within a 2000-character limit typical of Grant Application Forms. These statements were anonymised and given to four members of the group not involved in the costings exercise, but who represented funding bodies or sat on funding panels. Informal feedback was elicited about the content and style that was most helpful for allowing funders to judge the appropriateness of the resources being requested.

### Role delineation

The objective of this exercise was to describe variation between CTUs in the allocation of particular CTU activities to staff roles. We assessed variation across CTUs represented by members of the group on the involvement of each role in each of 163 activities which would be necessary in a clinical trial of an investigational medicinal product. This list of activities had been drawn up by the UK Trial Managers Network in 2009 (Additional file 2). Activities were entered as the first column of a spreadsheet. The exercise required CTU representatives to insert their own job category titles across the top row – taking as many columns as needed – and, for subsequent rows, ticking whether each job category was involved in carrying out the task in column A. Respondents could tick more than one job category for a particular activity as many activities within trials are multidisciplinary.

We did not specify job titles a priori because we already had a large volume of anecdotal evidence that: (1) job titles differ from unit to unit, (2) where units have similar job titles, it is unclear whether the roles are in fact the same and (3) not all units employ, for example, people called ‘programmers’ or ‘statisticians’ (minutes, 20 March 2013). Therefore, we allowed units to specify their own job titles/roles and, when they generally allocated activities in this way, to complete the form by allocating tasks to job families rather than discrete job categories per se – a shortcoming discussed below. By job families, we mean groupings of CTU workers, whose task sets are subjectively thought to be closely related in functional terms. For instance, one unit considered their trial manager, trial coordinator and senior trial manager, all to be working in ‘trial management’ (minutes, 20 March 2013). The most common job family terms across units were ‘statistics’, ‘study/trial management’, ‘data management’, ‘quality assurance’ (QA) and ‘clerical/admin’, with other job families, such as programming, not adopted by all units.

For each role, we used STATA v13 software to calculate Cohen’s *κ* (kappa), a chance-corrected statistic in which a value of 1 corresponds to perfect consistency, 0 to agreement no better than chance alone, and negative values to agreement worse than chance (STATA v13) [43]. We used Altman’s benchmark scale to assess inter-rater reliability [44]. A first analysis used a dataset in which the roles defined by the CTUs were informally mapped to their closest perceived equivalent roles at an index unit – the analyst’s home unit, selected for convenience. We then applied formal statistical tests of task-set agreement (see columns B to J, Additional file 3). A number of roles at the units, for instance ‘operations director’, had no obvious equivalents across the majority of units and so were left out of the analysis. A second analysis combined certain roles into job families for those units which had not presented data in this way; this analysis was designed to account for fuzziness at the boundaries of related roles that was reported by some units. The following roles were combined: administrative and clerical; senior and junior statistician; study manager and research assistant.

## Results

### Variation in costs

Summary results of the cost-comparison exercise are displayed in Table 1. At four CTUs, standard practice was to smooth the cost of variable across the study period, which made budgetary projection easier; at one CTU (A), variable costing was standard and has been smoothed for the purposes of this report, except where stated. Across the five CTUs, the median full economic cost of CTU activity was £769,637 (range: £661,112 to £1,383,323). Variation in overall costs were related to whether, as well as how, particular activities were costed; for instance, CTU D does not employ its own statisticians, meaning that one could expect to see an additional cost for non-CTU personnel to provide the statistical resource required for the trial. Across other CTUs, the median cost of senior plus junior statisticians (not including statistical programmers where used) was £76,799 (£61,035 to £103,755).

**Table 1:** Summary comparison of Clinical Trials Unit (CTU) costs for identical protocol

Trials unit	100%FEC D.A.	D.I.	Total staff costs (%)	Non-staff costs (%)	Indirect costs (%)	Total FEC	Total no. of FTEs attracting overheads	Total number of FTEs
A	£0	£362,350	£362,350 (55)	£65,375 (10)	£233,387 (35)	£661,112	0.89	2.00
B	£53,608	£443,924	£497,532 (36)	£66,150 (5)	£819,641 (59)	£1,383,323	0.43	3.00
C	£0	£638,611	£638,611 (54)	£131,776 (11)	£411,684 (35)	£1,182,071	2.00	3.00
D	£0	£364,042	£364,042 (52)	£39,914.02 (6)	£301,412 (43)	£705,368	1.43^a^	1.47^a^
E	£0	£574,701	£574,701 (75)	£82,550 (11)	£112,386 (15)	£769,637	0.3	2.49

^a^ CTU D did not include costs for statistical design and analysis, as they used external statisticians

*D.A.*directly allocated, *D.I.*directly incurred, *FEC*, *FTE* full time equivalents

The greatest variation between CTU costs was observed in the level of indirect costs (median £301,412, range £112,386 to £819,641), which accounted for between 15% and 59% of the full economic cost (median 35%). CTU B, whilst having median staff costs and the lowest non-staff costs, had the highest overall costs due to the relatively high level of indirect costs levied by its host institution (59% of the full economic cost). Excluding the CTU which did not provide statistical services at the time, the total number of FTEs ranged from 2.0 to 3.0; the number of FTEs attracting overheads ranged from 0.3 to 2.0.

A related issue was variation in the designation of project staff. When TRAC methods are applied (see above), HEI staff designated as researchers attract indirect costs whilst other HEI staff and NHS-based staff do not. The only roles which were consistently designated research staff were the senior trial methodology advisory roles (typically provided by a senior academic) and statisticians, although it was rare for study managers and research assistants to be on technical contracts. Data managers and programmers were typically, but not always, on technical contracts, with QA officers equally likely to be designated as researchers or not.

The second largest source of variation was the differences in staffing – although there were considerable commonalities in the way some roles were costed. Several over-arching issues emerged from interviews and from discussions at teleconferences and meetings. These are described first, followed by variation in staffing by job family; in this section, annual salary costs are described.

#### Over-arching issues

Estimating the direct costs of a trial for a grant application is an inexact exercise that tries to anticipate the resources required based on specific trial factors (setting, number of centres, sample size, recruitment rate, duration of intervention, number of follow-up visits and total duration of follow-up, outcomes to be measured, etc.).

The resources required for a trial are not constant over time but are difficult to schedule since trials may not run according to plan. Therefore, CTUs are typically project-managing a specific trial (and budget) in the context of a larger envelope of resources (and budget) accruing over a portfolio of trials. This justifies budgeting for an average amount of resource for a job family of activity for a trial and managing fluctuations in workload across multiple trials. CTUs with larger portfolios may be able to achieve this flexibility more easily.

CTUs have markedly different histories and infrastructure affecting staff-mix, established ways of working, portfolio size and activities they offer (potentially devolving some activities outside the unit). These factors are largely fixed but inevitably impact on the ways they manage and budget for trials.

CTUs varied in their access to NIHR CTU Support Funding (partly related to size and history), to charitable ‘infrastructure’ funding and to ‘core’ investment (directly by HEIs because trials generate large amounts of income through staff funded from other sources, such as medical charities, but able to collaborate on trials, etc.). NIHR CTU Support Funding is intended to be used to facilitate the submission of more, higher-quality grant applications to the NIHR but this and other resources, not derived from grants, allow CTUs/HEIs flexibility in costing grant applications. Core investment (e.g. research fellowships) may cover staff time, providing flexibility for a person to carry out research within a broad designated health area (e.g. disease-specific cancer; cardiovascular disease; arthritis).

#### Variation in staff costs by activity job family

Study management oversight by senior trials unit staff was mainly at 5% throughout – with one CTU dropping provision to 2% for the final 6 months and one CTU opting for 10% throughout; the base salary for this role, however, varied from £45,053 (often called a ‘research fellow’) to a professorial level, although salaries in the range £47,787 to £53,765 (‘senior research fellow’) were most common. These individuals generally played a role in proposal development and costing in the pre-award phase, and, post award, supervision of study staff and essential documentation, attendance at Trial Management Groups, Trial Steering Committees and Data Monitoring and Ethics Committees and interpretation and writing of trial reports. Some had substantial core funding from a variety of sources and some were wholly funded from grants.

Project management was much more complicated and heterogeneous with a wide variety of job titles and role boundaries making direct comparison difficult (see below, ‘Role delineation’). The project manager would typically be full time and have a salary in the range of £28,972 to £36,661, with some CTUs capping salaries for this work at just over £34,000. One CTU made considerable savings over the course of the project by variable costing of study managers, dropping the post from 100% to 40% during the recruitment period once all centres were open and, further, to 20% during the period in which patients were only being followed up. The representative of this CTU acknowledged that their unit benefitted from dependable core/infrastructure funding and economies of scale not available to many smaller units. These circumstances meant that, in the event of unexpected problems with governance or attrition which can take up time in the later phases of a trial, there were always staff available to deal with these problems effectively without including a costing to cover this risk for individual trials:

‘For a lot of units it is very difficult to have that fluctuation in work load within a project… it is about the infrastructure and the set-up that you’ve got as a unit’.

Project management staff were not limited to the project manager. One CTU budgeted for 25% of a more senior project manager (salary in the range of £37,756 to £45,053) in addition to the project manager themselves. It was more common to see a proportion of a more junior researcher, sometimes called a ‘research assistant’, to support the project manager.

Reassuringly, the level of funding for statistics varied little between units. Aside from unit D (see above) statistical support (excluding programming) was commonly at 30–33% throughout, with one unit preferring variable costing (20% throughout, and 100% in the last 6 months) of a junior statistician, usually salaried between £30,728 and £36,661. The junior statistician was typically supported by at least one senior statistician, typically at 5% and with a salary in the range £42,476 to £53,765, although occasionally the cost of this resource implied that senior statistical oversight would be carried out by someone on a much higher, professorial salary. Smoothing of junior statistician salaries across grants was near ubiquitous, despite more or less predictable peaks and troughs in activity (drafting the statistical analysis plan, reports for oversight committees, interim analyses, final analyses and write up).

The following rationale was given by one CTU representative and accepted as legitimate by funding-body representatives:

‘What CTUs are trying to do is to run a portfolio. Within that, you recognise that, despite your best intentions, trials have a life of their own. They very rarely go according to the timeline that you set, they very rarely go according to the budget that you set, and nearly all of the funding is fixed. So, you have to have 30% of a junior statistician. The point about burnout of the junior statisticians is incredibly important. If you keep loading them with four to six studies and expect them to deliver excellence at a relatively early part of their career, there can be psychological harms and professional costs’.

Two CTUs did not budget for clerical officers and, in each case, allocated what other CTUs saw as clerical tasks (such as site file composition, minute-taking and the processing of expenses claims) to research staff on higher salaries. For one unit this was a choice; for the other it was imposed on them by a host institution because of perceived imbalances in the departmental staffing profile (see above regarding designation of staff as researchers or not and the impact on indirect costs):

‘A lot of our trial managers spend a lot of time being very expensive administrators and secretaries so the only way we seem to be able to address that is to significantly increase the amount of assistant trial manager time’.

When included, clerical salaries ranged from £15,456 to £21,597 and such staff were never designated as researchers (and, therefore, did not attract indirect costs). Those at the bottom end were typically associated with data entry tasks and those at the top end were associated with tasks allocated to research staff at other institutions (site file composition, minute-taking, reimbursement of cost claims, and budgetary support).

Data managers, typically on nonresearch salaries of between £28,132 and £36,661, were budgeted at an average of 30% throughout most of a study, with some units varying the amount of time for this role across the duration of the trial. In some CTUs the boundaries between data management, data entry (more typically a junior research or clerical post) and programming were often ‘fuzzy’. Some units assumed that research nurses would input data on site (thus, a site cost rather than a CTU cost), whilst others arranged for paper Case Report Forms (CRFs) to be returned to the CTU for data entry. One unit outsourced its programming for data management, showing this activity in the budget as a consultancy fee instead of a staff cost. At other units, there was great variation in how programmers were costed in terms of their salary ranges and the duration of their input on the trial – with many costing this role only for 3 to 6 months in the first year and some smoothing the costs throughout. There was also some overlap in duties between database programmers and statistical programmers at some units, all of which made comparison difficult.

Quality assurance was an activity which was budgeted for by three of the five CTUs. One CTU funded this activity wholly from core/infrastructure funding; another had never been able to fund a formal QA role from either core or grant funding. A number of CTUs reported distributing key QA tasks, such as risk assessment and the authorship/update of standard operating procedures, across their staff, because they were unable to fund a QA officer adequately. Where present, QA officer salaries were in the range of £21,597 to £34,575, with higher whole time equivalents (WTEs) requested for junior (e.g. 20%) compared to senior staff (e.g. 5%). QA officers were sometimes designated as having academic and sometimes nonacademic contracts.

A breakdown of non-staff costs can be found in Table 2. In two CTUs, travel costs associated with oversight (Trial Steering and Data Monitoring) Committees (although many units were increasing the use of teleconferences) and, particularly, on-site monitoring accounted for two thirds of all non-staff costs, compared to between 19% and 30% of non-staff costs in other units. Most units reported an increasing shift towards central monitoring although some are still required by sponsors to perform the majority of their monitoring on site. Some sponsors still require on-site monitoring, partly because of concern about the safety and effectiveness of central monitoring, even in ostensibly low-risk studies and despite contrary expert opinions [9, 45, 46].

**Table 2:** Non-staff costs

Trials unit	A	B	C	D	E	£ median (range)	Median (range) % of non-staff costs
Travel	£19,380 (30%)	£12,750 (19%)	£88,190 (67%)	£25,952 (65%)	£17,004 (21%)	£19,380 (12,750 to 88,190)	30 (19 to 67)
Equipment	£1,500 (2%)	£2,600 (4%)	£1,800 (1%)	£ 0 (0%)	£0 (0%)	£1500 (0 to 2,600)	1 (0 to 4)
External consultancy	£18,145 (28%)	£3,000 (5%)	£28,120 (21%)	£7,750 (19%)	£55,544 (67%)	£18,145 (3,000 to 55,544)	21 (5 to 67)
Consumables	£25,850 (40%)	£47,800 (72%)	£13,666 (10%)	£6,212 (16%)	£10,002 (12%)	£13,666 (6,212 to 47,800)	16 (10 to 72)
Total	£65,375	£66,150	£131,776	£39,914	£82,550	£66,150 (39,914 to 131,776)

‘… there’s always some rogue sites… and you can’t underestimate the value of contact… for keeping the study in the forefront of people’s minds.’

For the three units using proprietary, fee-based, electronic data capture systems (complementing, or instead of, in-house data management staff), the fees were £5,500 (CTU A: not including £31,697 programmer costs), £7,500 (CTU D: not including programming undertaken as part of the work of 0.3 WTE data managers) and £45,640 (CTU E: not including £7,955 programmer costs). CTU E’s costs included a database licence at £10,000 and £35,640 (£33 per site, per month) for site access to the electronic CRF (eCRF). The use of an eCRF with this cost structure is not something for which the other CTUs had to cost. CTU A used paper CRFs at the centres, returned by post, resulting in low database costs and above average consumable costs. For commercial database systems with a flat fee workplace licence, units with larger portfolios were able to achieve greater economies of scale. Two units used programmers to adapt or modify locally developed systems: CTU B at £59,252 and CTU C at £23,800 – although, as with CTU D, this figure masked a certain amount of additional programming undertaken by data managers.

### Justification of costs

Representatives of four CTUs submitted anonymised statements justifying the costs they had submitted in the exercise described above (Additional file 4). Representatives of funders preferred a written justification of costs that combined details of FTEs with indicative tasks for given roles. They rated as most useful the submissions from CTU A and CTU C, although the former lacked detail on non-staff costs and the latter lacked detail on the period of deployment of staff across the duration of the trial. Funders preferred these submissions because ‘they enabled a fuller understanding of the activity to be undertaken and enabled an informed assessment of the financial costs and value for money’.

### Role delineation

The units responded with an average of 7 job titles each, ranging from 3 to 11 job titles across the units. Once duplicates had been removed, there were 43 unique job titles (see Table 3). Nine of these were felt to map adequately across a number of units but, when tested formally, consistency in the allocation of activities to roles was fair (*κ* = 0.21–0.40) for senior statisticians and data managers and poor for other roles (*κ* < 0.20) (Table 4; Additional file 5). When we combined roles into job families, consistency in the allocation of activities to roles was fair for statisticians and data managers and poor for other job families (Table 5). The role delineation exercise confirmed that micro-costing at the level of the individual task is not feasible and that, for pragmatic reasons, costing has to be taken at the level of the role or job family, with the potential to vary or smooth costs according to anticipated workload across different periods of the study (minutes 15 July 2013).

**Table 3:** Clinical Trial Unit (CTU) job titles as submitted by nine CTUs

CTU	Job titles submitted
Sheffield	QA
	Trial Manager
	Data Manager
	Senior Statistician
	Junior Statistician
	RA
	Administrator
	CTU Advice
	Clerical
Birmingham PC-CRTU	Quality Assurance Team
	Trial Management
	Data Manager
	Statistics
	Programming
CTEU Bristol	Unit director
	Unit manager
	Research fellow
	Research associate (coord)
	Research assistant (coord)
	Assistant coordinator
	Administrator
	Statistican (RF)
	Statistician (RAassoc)
	Statistician (Rassis)
	Database manager
	Research sister
	Research nurse
Leeds CTU	Statistician
	Head of Trial Management, Senior Trial Manager, Senior Trial Co-ordinator
	Trial Co-ordinator /Trial Management Assistant
	Clinical Trial Associate
	Senior Data Manager
	Data Management Assistant / Data Entry Clerk
	Information Systems (programmer)
MRC CTU	Project Lead
	Programme Lead
	Senior Statistician
	Junior Statistician
	Trial Physician
	Clinical Projects Manager/Clinical Operations Manager
	Trial Manager
	Data Manager
	Trial Assistant
	(Senior) analyst programmer
	Clinical Data Systems Manager
	Data scientist
	Research administrator
Newcastle CTU	Statisticians
	Data managers
	Senior Trial Manager.
	Trial Manager
Oxford CTSU	Chief & Principal Investigators
	Statistician
	Trial Coordinator
	Unit administrator
	Contracts specialist
	Clinical fellow
	Training & monitoring coordinator
	Monitors
	Supplies coordinator
	Trial Manager
	Trial administrator
	Senior System Developer
	Laboratory manager
	Validation coordinator
	Database administrator/IT support
	Health economist
Penninsula CTU	Statistician
	Trial Manager 1
	Trial Manager 2
	Data Manager 1
	Data Manager 2
	Data Programmer
	Data Assistant 1
	Data Assistant 2
	Unit Coordinator
	Trials Secretary
Southampton CTU	Statistician/s
	Senior Clinical Trials Manager
	Clinical Trials Manager
	Clinical Trial Coordinator
	Data Manager
	Data Officer
	Clinical Trials Assistant
	Quality & Regulatory Manager
	Quality & Regulatory Officer
	Clinical Research Fellow
	Operations Director
	Clinical Trials Unit Administrator

**Table 4:** Consistency in role definition – roles as submitted

Role	Consistency (kappa)
QA	0.01
Trial manager	0.14
Data manager	0.30
Senior statistician	0.36
Junior statistician	−0.01
RA	0.02
Administrative	0.1
CTU advice	0.09
Clerical	0.11

*CTU* clinical trials unit, *QA* quality assurance, *RA* research assistant

**Table 5:** Consistency in role definition – roles combined in job families

Role	Consistency (kappa)
QA	0.01
Trial manager/RA	0.18
Data manager	0.30
Statistician	0.35
Administrative/clerical	0.12
CTU advice	0.09

*CTU* clinical trials unit, *QA* quality assurance, *RA* research assistant

## Discussion

### Summary of findings

This study confirms the subjective impression of grant award panel members that CTU costs on grant applications vary in ways that cannot be attributed wholly to study design or complexity (held constant in this exercise by use of the trial vignette). Levels of indirect costs levied by HEIs, level of staffing requested (often related to the size of the unit and its access to non-project-specific funding, variably referred to as core/infrastructure funding), number of WTEs attracting overheads and level of site-based monitoring activities were the main drivers of variation in CTU costs.

### Strengths and limitations of the study

The diverse perspectives of contributors to this study (representing funders, members of grant award panels as well as CTUs) is an important strength. CTU members were chosen (from a larger number of nominees) for the group on the basis of their stated expertise with respect to costing research. Representatives of participating units found no difficulties in costing the protocol and, whilst noting differences in definition and attribution of costs for the set-up period of a trial, confirmed that other timescales were within what was considered normal locally. The conduct of this exercise over 12–18 months, with several opportunities for debate and feedback, is another strength.

The main limitation of this exercise is the limited sample size. The findings of this paper reflect the pooled experience of members from only ten of the 45 CTUs registered with the UK Clinical Research Collaboration in 2013. Although this sample of CTUs is diverse in terms of funding, portfolios and organisation, it is unlikely that they are fully representative of all UK units. In particular, the comparative costs exercise is based on detailed feedback from a self-selected sample of just five CTUs that were able to prioritise an unfunded contribution to this exercise, fewer than considered satisfactory for thematic saturation [47]. Our remit did not permit a detailed discussion of the impact of access to core infrastructure on the costs and activity of any particular CTU, although the group felt that such access had an important impact on research costs (minutes, 20 March 2013, 15 July 2013, 2 October 2013 and 9 October 2013).

A second limitation is the extent to which costing of the vignette by a CTU followed processes that would have been used when costing an actual application for funding for a trial. The formatting of the costs that were reported by CTUs and details provided in subsequent interviews demonstrated that the costs had been generated by staff familiar with CTUs’ usual costing processes and experienced in applying them. However, the exercise could not reproduce all of the formal steps for an actual grant submission and it is possible that further checks, required as part of approving a grant for actual submission, might have led to some revisions.

### Findings in the context of previous trial literature/discussion of findings

Our findings confirm that, for some trial activities – for instance statistical support – there is remarkable similarity in approaches to costing between CTUs. Also, despite the differences in headline CTU costs, even the lowest CTU costs exceeded £0.5 m. We mention this figure because some funding opportunities invite applications for nonpilot trials and, nevertheless, specify this, or a lower figure, as a funding ceiling; specifying a ceiling in this way tends to lead to trial budgets just under the ceiling but which are not credible.

Similarity may arise either because the underlying costs of an activity are, indeed, similar across CTUs or just because CTUs have adopted a similar model for costing – potentially, communicated from one CTU to another because ‘that’s what the funder will accept’. The extent to which CTUs cost planned research at the level of the task was discussed, and was observed to vary; some did this (albeit, not with the granularity a CRO would use) and others relied on standard costing templates in which many elements can vary only at the margins from project to project. Several members argued that task-based costing is difficult and time-consuming to do, and not necessarily predictive of the actual work required on a project.

This study has begun the process of documenting real differences in the activities and associated costs which CTUs feel are necessary to deliver a particular trial, and how they express those requirements. The differences in role nomenclature (see Table 3), the scale at which different units report allocating activity and costs – at the level of the job family (e.g. study management), the role (e.g. study manager or research assistant) or the task (e.g. site-monitoring visit) – presented a barrier in comparing role delineation and task allocation between units. By allowing some CTUs to complete this exercise using discrete job categories, and others using job families, the responses may reflect disparate staff profiles. Even if this could be ignored, further validation of the data would be required before meaningful conclusions on comparative role delineation can be drawn. For instance, six out of nine units responding to the exercise did not declare that any tasks from the list were associated with a quality assurance (QA) officer, although in at least one case this is because they had never been able to fund a formal QA role from either core or grant funding.

The findings provide an empirical basis for what was reported anecdotally at the outset. First, there are local idiosyncrasies in resource need, estimation and allocation. This should be unsurprising given the different histories behind CTUs with some of the oldest coalescing around one or more clinical investigators in a particular disease area, and newer general-purpose or region-specific CTUs often being created. For instance, most of the trials managed by one CTU are collaborations with ‘in-house’ chief investigators who are closely involved in many aspects of their trials (see Additional file 3). In many other CTUs, collaborations with external, ‘client’ chief investigators are the norm, with more responsibility for trial management falling on senior CTU staff.

Second, the demands of project implementation fluctuate in real life unpredictably, more than can be reliably estimated at the planning stage; maintaining fuzzy boundaries between roles allows less well-resourced units to respond flexibly to project implementation, given the capacity and expertise they have available at a particular time. When compared, superficial differences in staff costs sometimes appeared to even out in, within and between job families. For instance, an employee with a particular role at one CTU might have a relatively high salary but be costed for a smaller proportion of time for the relevant job family at another CTU.

### Implications

These findings have implications for funders, HEIs and CTUs. Should funders care about variations in costs, or simply make a judgement about perceived value for money? The answer may differ by funding opportunity, since commissioned research topics usually attract competing bids whereas researcher-proposed topics do not. More fundamentally, judging value for money becomes much more difficult if HEIs cross-subsidise CTUs or impose financial constraints. When judging whether CTU costs in a grant application are appropriate, funders should bear in mind that most direct costs are requested for good reason, e.g. to support activities required by sponsors, and are relatively standard across CTUs (although the activities required by sponsors may vary, e.g. in relation to monitoring). CTUs should strive to provide as much information and detail on costs as possible to ensure that funders have a transparent view.

The largest variations in costs are outside the control of CTUs, arising from factors that are features of, or are imposed by, host organisations. These include staff profile, levels of indirect costs and access to core funding. Levels of indirect costs have also attracted attention in the USA where, between 2003 to 2012, indirect costs of National Institute for Health-funded studies increased by 16.9% compared with 11.7% for direct costs [48]. Calculation of indirect costs in the UK is complex but is, ultimately, a function of choices made by HEIs when they interpret TRAC guidance. For example, an institution may require a grant budget to achieve a percentage threshold of ‘cost recovery’ (indirect income), which could be realised by assuming that researchers (who attract indirect income) rather than technical or administrative staff (who do not) will carry out necessary activities. HEIs can be penalised by the government for indirect costs which are above the upper quartile of sector rates [40]. Finance officers at HEIs must approve grant applications at the time of submission; this requirement means that an HEI can, in effect, prevent the submission of an application if the HEI considers that the research will not cover its costs [49].

Local idiosyncrasies of host institutions (minutes, 20 March 2013), as well as the diverse portfolios of CTUs (minutes, 15 July 2013), make the adoption of a common, standardised costing template impossible. In practice, CTU-based researchers costing grant applications expect the budget to reflect the needs of specific studies (minutes, 15 July 2013). Workload models for organisations have principally been published by teams working within the US National Institutes of Health, National Cancer Institute and the National Cancer Institute of Canada; these are primarily designed to estimate site costs (more similar to templates increasingly adopted by NHS hospitals to estimates sites costs), not CTU activities. This literature supports the view that trials are increasing in complexity but this conclusion may be influenced by the predominant focus on cancer trials. Workload modes are criticised for over-simplicity and are implicated in staff burnout and poor quality standards [34, 36].

These criticisms resonate with researchers in CTUs with responsibility for estimating trial management workload. Many trials commissioned by the UK NIHR involve difficult-to-reach study populations and complex interventions, making trial management workload unpredictable [34]. CTU representatives in our group knew of costing templates that had been developed over years and yet which do not meet the needs of the trial team any more reliably than costs generated by an experienced proposal developer (minutes, 20 March 2013). It was also noted that, when preparing a budget, a CTU starts with a staff profile which cannot be changed quickly and easily to suit the introduction of a standardised costing template (minutes, 20 March 2013). Recent attempts by three neighbouring CTUs to build common approaches to costing were reported to have failed for this and other reasons (minutes, 20 March 2013). Consequently, CTUs involved in this study reported relying on informal frameworks to modify staffing by the scope, scale and stage of the trial (minutes, 15 July 2013).

Projecting a timetable and recruitment for a trial are critical to estimating a trial budget and are often uncertain. There was scepticism among our group members that national targets to bring down approval times were uniformly successful [20], or robust to subversion [50–53]. The system for attributing costs in NHS R&D [54] often resulted in long delays in the initiation of recruitment at participating sites [18]. Delays at this early stage quickly lead to a shortfall in recruitment. The planning fallacy, that is, the tendency for people to ‘underestimate the time required to complete a project, even when they have considerable experience’ [55], also plays a role in the underestimation of variable costs, as a smaller than anticipated fraction of patients screened typically enrol in a study [56–59].

Despite growing evidence that an increase in central monitoring and reduced site monitoring can safely reduce costs [45, 60–63], some sponsors and CTUs still have reservations that central monitoring can reliably maintain Good Clinical Practice (GCP)- and protocol-compliance. Senior CTU staff who are writing proposals should consider training in risk-based monitoring and research should be considered which assesses the barriers to, and facilitators of, uptake and implementation of leaner monitoring strategies.

## Conclusions

Some variation in costs is due to factors outside the control of CTUs such as access to core funding and levels of indirect costs imposed by host institutions. CTUs need to undertake a clear and transparent costing that fully explains the resources required when submitting a grant application to enable funders to make informed decisions. Research is needed on barriers to implementing evidence-based strategies which minimise costs, especially, risk-based monitoring strategies.

## Additional files

Additional file 1: Example protocol for costing exercise. (DOCX 13 kb)

Additional file 2: Generic clinical trial tasks (UKCRC TMN). (DOCX 23 kb)

Additional file 3: Tasks by role – all CTUs. (XLSX 129 kb)

Additional file 4: Justification of costs statements. (DOCX 18 kb)

Additional file 5: Summary of tasks by role by CTU. (XLSX 20 kb)

## Abbreviations

CTU: Clinical Trials Unit
eCRF: electronic Case Report Form
GCP: Good Clinical Practice
HEFCE: Higher Education Funding Council for England
HEIs: Higher education institutions
NIHR: National Institute for Health Research
TRAC: Transparent Approach to Costing
UKCRC: UK Clinical Research Collaboration
WTE: Whole time equivalent
