# Supplementary material for: Comparative costs and activity from a sample of UK clinical trials units
Source: Trials. 2017 May 2;18:203. doi: 10.1186/s13063-017-1934-3 (PMC5414193; doi:10.1186/s13063-017-1934-3)
Supplement: Supplementary file 1 — Example protocol for costing exercise. (DOCX 13 kb) [file 13063_2017_1934_MOESM1_ESM.docx]

OBJECTIVES

To compare the clinical and cost-effectiveness of the [Intervenion 1] and [Intervention2].

PROPOSED DESIGN

Parallel-group individually randomised controlled trial with internal pilot trial to assess feasibility.

SETTING

Twenty acute hospital trusts.

TARGET POPULATION

Inclusion criteria: presentation with [condition]; age = 18 years; signed informed consent; able to undergo general anaesthesia. Exclusion criteria: 5. Previous repair of [condition].

INTERVENTIONS

(1) [Intervenion 1]; or, (2) [Intervenion 1].

OUTCOMES

Primary outcome: self-reported recurrence of [Intervenion 1], data collected by research nurse over telephone at 24 months. Secondary outcomes (assessed at 0, 3, 12 and 24 months, except where indicated): [Outcomes removed for academic-in-confidence purposes].

SAMPLE SIZE

A logrank test using Simpson’s rule allows for varying patient follow-up (min 2 years, max 4). Based on [a previous study] we assume 15% control arm recurrence within 1 year and 5% p.a. thereafter. We postulate these rates dropping to 5% in year 1 and 2% per annum thereafter. With 20% loss to follow-up, we require 130 per arm for 90% power and 2alpha=5%.

PROJECT TIMETABLE

We propose a 66 month (m) study: 12m setup; 24m recruitment; 24m follow-up; 6m close-out, analysis and write-up.

FEASIBILITY

Audits indicate 35 eligible patients per year at Oxford, 20 elsewhere. We assume that: (1) 4 centres per m. will open from m8, with 20 open by the end of m12; (2) due to surgery lead times, operations on randomised patients will not start until m13, with all centres operating on randomised participants by end of m17; (3) 2.9 eligible participants per m. are available at [lead centre], 1.3 at other centres; (4) that 45% of eligible participants will consent and survive the enrollment period to randomisation and surgery. By the end of m18 we should have initiated 20 centres and operated on 50 randomised participants. A feasibility assessment at 18m will employ these stop/go criteria: (1) initiation of no fewer than 15 centres; (2) operation per protocol on no fewer than 30 randomised participants.
