# Supplementary material for: Comparative costs and activity from a sample of UK clinical trials units
Source: Trials. 2017 May 2;18:203. doi: 10.1186/s13063-017-1934-3 (PMC5414193; doi:10.1186/s13063-017-1934-3)
Supplement: Supplementary file 2 — Generic clinical trial tasks (UKCRC TMN). (DOCX 23 kb) [file 13063_2017_1934_MOESM2_ESM.docx]

|  | **Funding & Grants** |
| --- | --- |
| 1 | Literature review |
| 2 | Develop research question |
| 3 | Liaison with statisticians for design proposal and sample size |
| 4 | Scoping of requirements (staff and research costs) |
| 5 | Preliminary discussions on sponsorship arrangements |
| 6 | Costing / FEC |
| 7 | Negotiations with institution that will manage / receive funding |
| 8 | Completion & submission of grant application form |
| 9 | Grant Interview / Presentation |
| 10 | Revisions to proposal as required |
| 11 | Acceptance of offer and completion of contracts |
| 12 | Parallel / additional submissions to other funders |
| 13 | Confirmation of Sponsorship arrangements |
|  |  |
|  | **Monitoring & pharmacovigilance** |
| 14 | Development of trial monitoring plan |
| 15 | Assemble DMC |
| 16 | Assemble TSC |
| 17 | Declaration of Interests |
| 18 | Development of charters / procedures for oversight committees |
| 19 | Development & approval of trial specific pharmacovigilance SOPs and forms |
| 20 | Development of monitoring schedule, SOPs, forms |
| 21 | Preparation and submission of regular reports to DMC & TSC |
| 22 | Preparation and submission of annual safety report |
| 23 | Scheduling & facilitating Data Monitoring Committee meetings |
| 24 | Scheduling & facilitating Trial Steering Committee meetings |
| 25 | Site Monitoring visits |
| 26 | Preparation of monitoring reports |
| 27 | Review of monitoring reports |
| 28 | Follow-up & resolution of monitoring issues |
|  |  |
|  | **Finance & HR** |
| 29 | Negotiations with institution that will manage / receive funding |
| 30 | Set-up of cost centres, budget signatories, etc. |
| 31 | Liaison with HR for JDs and pay scale ratings for trial specific staff |
| 32 | Advertising for trial specific staff |
| 33 | Interview of trial specific staff |
| 34 | Appointment of trial specific staff |
| 35 | Training of appointed staff |
| 36 | Training of existing coordinating staff |
|  |  |
|  | **Protocol & procedures** |
| 37 | Development, review and approval of the following: |
| 38 | Protocol |
| 39 | PIS |
| 40 | Consent Form |
| 41 | SOPs |
| 42 | Forms |
| 43 | Case Report Forms |
| 44 | Printing of CRFs |
| 45 | Printing documents for site files |
| 46 | Assembling site files |
| 47 | Developing new questionnaires or licensing use of existing instruments |
| 48 | Development and validation of randomisation procedure & related SOPs for site staff |
|  |  |
|  | **Suppliers & contractors** |
| 49 | Negotiation with labs, outside contractors |
| 50 | Audit of supplier e.g. labs, IT developers, IMP suppliers |
| 51 | Confirmation of suppliers, labs etc. |
| 52 | Agreement of terms with suppliers / contract labs |
| 53 | Review and completion of contracts as required |
| 54 | Collect pricing and timeline information on consumables |
| 55 | Order consumables (Site files, blood collection tubes, measurement equipment etc) |
|  |  |
|  | **Study sites** |
| 56 | Negotiations with potential sites regarding interest and feasibility |
| 57 | Costing & feasibility at site |
| 58 | Submission of applications for research governance approval |
| 59 | Development of training for site investigators |
| 60 | Dispatching equipment to sites |
| 61 | Schedule initiation visits / training days |
| 62 | Forward details of approvals to site |
| 63 | Write to sites to confirm recruitment can begin |
|  |  |
|  | **Applications** |
| 64 | Draft, review, approve, sign and submit applications and amendments to |
| 65 | Ethics |
| 66 | MHRA |
| 67 | Other regulators (PIAG, GTAC, HTA_ |
| 68 | Receive, manage and circulate approvals from regulatory bodies |
| 69 | Liaise with regulators to meet terms of approval throughout study period |
|  |  |
|  | **Advertising & promotion** |
| 70 | Draft, review and approve details of: |
| 71 | Media announcements |
| 72 | Advertising |
| 73 | Study Website |
| 74 | Study Newsletters (Patient & Site) |
|  |  |
|  | **Data management & related activities** |
| 75 | Third party audit if supplier being used for database development |
| 76 | Development & approval of User Specification |
| 77 | Development and approval of Functional Specification for Database |
| 78 | Development and design of database |
| 79 | Qualification and testing of database |
| 80 | Development of specific data management SOPs |
| 81 | Receive data |
| 82 | Chase outstanding data |
| 83 | Check data & raise queries |
| 84 | Enter data |
| 85 | Temporary database lock |
| 86 | Data extracted for periodic reports |
| 87 | Final database lock |
| 88 | Backup |
| 89 | Key Activities in the Conduct of a Clinical Trial |
| 90 | Archiving |
|  |  |
|  | **IMP related activities** |
| 91 | Identification of IMP manufacturer / supplier |
| 92 | Collection of key documents from IMP manufacturer for submission to regulators |
| 93 | Investigator's Brochure (development / approval / circulation) |
| 94 | Identify QP |
| 95 | Agreement of contract for the supply of IMP |
| 96 | Develop and approve label |
| 97 | Consider storage requirements e.g. RT, refrigerator |
| 98 | Purchase of temperature control devices / agreement of logistic issues for transport |
| 99 | Manufacture, release and delivery of batches |
| 100 | Provision of QP release paperwork to sites |
| 101 | Re-order of IMP throughout trial |
|  |  |
|  | **Pharmacy** |
| 102 | Development and approval of SOPs and forms |
| 103 | Prescriptions |
| 104 | Dispensing record |
| 105 | Return |
| 106 | Ordering |
| 107 | Re-call |
| 108 | Destruction |
| 109 | Accountability |
| 110 | Staff training |
| 111 | Monitoring storage conditions |
|  |  |
|  | **CTU activities after enrolment of first patient** |
| 112 | Randomisation |
| 113 | Drug dispatch |
| 114 | Results reviewed & returned to sites (e.g. safety blood samples) |
| 115 | Central SAE collection |
| 116 | SAE review & classification |
| 117 | Expedited reporting of SUSARs or safety issues |
| 118 | Annual safety report - MHRA |
| 119 | Annual progress report to REC, Funding bodies, Sponsor etc |
| 120 | Review of reports |
| 121 | Liaising with sites regarding deviations |
| 122 | Submission of amendments |
| 123 | Re-training of staff |
| 124 | Budget & cost review |
| 125 | Ongoing liaison with finance department regarding costs. |
|  |  |
|  | **Site Activities** |
| 126 | Assist in preparation of applications for local approvals |
| 127 | Staff training and initiation |
| 128 | Collection of CVs |
| 129 | Complete delegation log / collect |
| 130 | Preliminary screening of patient lists / notes etc |
| 131 | Invited patients to attend screening visit |
| 132 | Consent patients |
| 133 | Study visit as per protocol |
| 134 | CRF completion |
| 135 | Registration of patients |
| 136 | Completion of prescriptions |
| 137 | Dispensing of study drug |
| 138 | Data collection and submission to Sponsor / Coordinating Centre (incl AE & SAE) |
| 139 | Drug accountability |
| 140 | Maintenance of site file |
| 141 | Submission of amendments to NHS organisation |
| 142 | Booking courier for transport of specimens |
| 143 | Writing to patients with appointment details |
| 144 | Clinic room booking / book scans etc. |
| 145 | Requesting hospital notes for visits |
| 146 | Notification of patients GP |
| 147 | Resolution of data queries |
| 148 | Assist with monitoring visits |
| 149 | Internal reporting to Trust |
| 150 | Archiving |
| 151 | Investigators meeting |
| 152 | Team meetings in relation to trial |
|  |  |
|  | **Statistics & Study Reports** |
| 153 | Development and approval of analysis plan |
| 154 | Data cleaning |
| 155 | Blind data review |
| 156 | Statistical analysis and review |
| 157 | Preparation of final study report for regulatory bodies |
| 158 | Reports to funders & other bodies |
| 159 | Development of paper for publication |
|  |  |
|  | **Study Archiving** |
| 160 | Site close-out visit |
| 162 | Validation or archiving arrangements |
| 163 | Archiving of material |
