# Supplementary material for: Comparative costs and activity from a sample of UK clinical trials units
Source: Trials. 2017 May 2;18:203. doi: 10.1186/s13063-017-1934-3 (PMC5414193; doi:10.1186/s13063-017-1934-3)
Supplement: Supplementary file 4 — Justification of costs statements. (DOCX 18 kb) [file 13063_2017_1934_MOESM4_ESM.docx]

TRIALS UNIT A.

The major expenditure is CTU staff costs associated with delivery of the trial. CTU staff are not included full time throughout but are apportioned subject to the requirements of the stage of the study. A Senior Trial Manager will project manage the study, ensure necessary central and local site approvals are in place at twenty sites, maintain the protocol and associated documents, finalise contracts, establish/maintain the oversight groups (TSC, DMEC, TMG), and support reporting requirements (ethics, sponsor and funder). The STM decreases to 0.4 FTE 6 months into the recruitment phase when the majority of sites will be established and to 0.2 FTE in follow-up, rising to 0.6 FTE for 6 months in analysis to facilitate site closeout and publications. A data manager is included full time for 3 months of set-up to develop the CRF’s, write the database, randomisation and reporting specifications, test the implementation and establish the data management processes for the trial. In recruitment they will send data queries, prompts and chase missing data and ensure high quality data and compliance. The FTE reduces to 0.4 in recruitment and 0.2 in follow-up and analysis as the intensity of incoming data deceases.  A data entry clerk/ clerical assistant is included to conduct data entry and support administrative requirements (organising meetings, chasing and circulating and chasing documents etc). This post starts at 0.2FTE for 3 months in set-up and rises in recruitment, follow-up and analysis to 0.4FTE. A medical statistician is included at 0.2FTE to review the CRF’s, randomization algorithm, database specifications, write and finalise the Statistical Analysis Plan and provide statistical monitoring and reporting for the trial and independent oversight groups. This increases to full time for the final analysis. They are supported by a principal statistician at 0.05FTE, rising to 0.1FTE for analysis. A programmer is included full time for 3 months to implement the randomization system trial database and reports, reducing to 0.1FTE in recruitment and follow-up to maintain the database, server and implement amendments. Non staff costs cover the cost of PPI input, travel to sites and oversight group meetings, trial specific printing, stationary, phone, fax and postage costs along with a contribution to storage, archiving and database licence costs.

TRIALS UNIT B.

Staff Costs:

The ***Statistician*** will be responsible for statistical design and analysis throughout, producing interim analyses and reports as required.  The CTU has a robust Quality Management System and a part time ***Monitor*** will be appointed to assist with Risk Assessments and Quality Management Plans and to provide on-site and in-house monitoring.  A part time ***Programmer*** will be appointed to develop and maintain the trial database.  A full time ***Trial Coordinator*** is essential to coordinate the trial and manage the project as a whole.  During the first year they will be responsible for finalising the protocol, securing regulatory approvals, and developing data collection tools and trial documentation (Trial Master File, Site Files *etc*).  Following this, they will be responsible for day to day management (pharmacovigilance, monitoring recruitment and data collection, problem solving, overseeing drug supply, producing reports and feedback to the oversight committees etc).  During the final year they will ensure timely validation, analysis and reporting of the findings in collaboration with the Trial Management Group.  It is important that the Trial Coordinator is supported by a part time ***Trial Administrator*** over this period.  There will be a large amount of data received over a relatively short period of time and this person is essential to ensure data is verified, queried and entered onto a database in a timely fashion.  All staff will be located within the CTU at the University of [XXX].

Non Pay:

On the basis of past experience we have estimated the costs of consumables based on contacting 300 patients (initial invitation letter and two CRFs per patient), miscellaneous office and general running expenses (general stationary, printing, photocopy, postage, telephones, filing cabinets).  Equipment includes PC’s for project staff and licences for software and quality of life, a printer, supply of toners and filing cabinets in which to hold the TMF.  We have estimated the number of journeys anticipated for patients at £2.50 per patient to cover the additional visits required.  Subsistence and travel for meetings of the oversight committees including Trial Steering Committee and Trial Management Group (up to 10 people twice a year).  The randomisation service is based on a calculated contribution to cover the central resource.

TRIALS UNIT C

CTU COSTS

• Trial Manager (1.0 WTE for 66 months): day-to-day running of study, designing study

documentation, ethical and research governance approvals, liaising with CI, site PIs and

methodological specialists, writing reports and initial draft of research papers, dissemination.

• Research Assistant (0.6 WTE for 36 months): Site-setup, monitoring visits.

• Data Manager (0.3 WTE for 63 months): web-based database and randomisation system, data

validation, cleaning and management.

• CTRU Advice (DH, 0.05 WTE for 66 months): Trial strategic advice.

• Admin/Quality Assurance (0.2 WTE for 60 months): Ensure delivery in accordance with CTRU and

regulatory requirements.

• Junior Statistician (0.3 WTE for 60 months): Preparing statistical analysis plan, conducting statistical

analysis, preparing data for the DMEC and TSC.

• Senior Statistician (MB, 0.05 WTE for 60 months): Statistical analysis.

• Clerical Officer (0.5 WTE for 66 months): facilitate meetings, purchasing, expenses, prepare

questionnaires and documentation.

• Junior Health Economist (0.5 WTE for 6 months)

• Senior Health Economist (AW, 0.05% for 60 months)

Non-staff costs on CTRU budget:

• Travel and subsistence – 11 TMGs, 11 TSCs, 1 HTA welcome meeting, 20 site set-up visits, 100

monitoring visits, conference attendance: £48110

• Site initiation and monitoring visits - 20 set up visits, 100 monitoring visits: £24000

• Teleconferences - 50 TMGs, 10 DMECs: £11680

• PPI: 2 representatives at TSCs: £4400

• Equipment – 3 computers: £1800

• Consumables (inc printing, promotional items, publications, site files, archiving): £13666

DATABASE

• 10 days DB set-up: £3500

• 58 months DB hosting and maintenance: £20300

• 28 months randomisation: £4320

TRIALS UNIT D

The funding we seek reflects the level of risk attached to the research following our combined risk assessment with the Sponsoring Trust. This study will be managed and monitored by *CTU with named staff from the Unit supporting the research team. On a day-to-day basis, sponsor-level activities will be carried out by *CTU, with reporting lines to the Sponsor who will ensure that all obligations have been adhered to. *CTU will set up the delegated responsibilities with the Sponsor on behalf of the trial team.

*CTU operates a blended model of trial management with a Senior Trial Manager who will line manage the Trial Manager and take responsibility for the projects’ progress and adherence to governance. The Trial Manager on the project will be supported by an Assistant Trial Manager. This frees up the TM from carrying out lower level activities (cost saving) and means greater project support in times of sickness and holiday cover.

*CTU will prepare the clinical trial protocol, information sheets and consent forms (questionnaires if applicable).

We will apply via for a favourable opinion from a Research Ethics Committee. We anticipate that site-specific assessments (IRAS SSI form) will be required in respect of each participating sites. The PI at site is responsible for completing the SSI and they will be supported by the trial manager throughout the process.

Post approval *CTU will prepare Investigator Site Files for each of the participating sites. As part of the site set up process we will arrange an initiation visit at which we would take the team members through their roles and responsibilities on the trial.

During the follow up period the study will require ongoing trial management so that safety data as required by the regulatory bodies are collected. The study will use the *CTU system for SAE reporting. A nominal cost of this is included. During the trial *CTU will submit safety reports to REC, R&D and the in addition to any reports the funder stipulates.

NCTU will initiate and monitor site close down. End of study reports will be completed and submitted with all the appropriate bodies.
